# Association of late-life blood pressure change with cerebral small vessel disease in the MIND-China study

**DOI:** 10.1186/s40001-024-01953-x

**Published:** 2024-07-18

**Authors:** Wei Lu, Qingping Ma, Jiafeng Wang, Chunyan Li, Qianqian Xie, Ziwei Chen, Huisi Zhang, Lin Song, Yifeng Du

**Affiliations:** 1 Department of Neurology, Shandong Provincial Hospital, Shandong University, Jinan, Shandong China; 2 Shandong Provincial Key Medical and Health Laboratory of Intensive Care Rehabilitation, Shandong Provincial Third Hospital, Cheeloo College of Medicine, Shandong University, Jinan, 250031 China; 3 Department of Neurology, Shandong Provincial Hospital affiliated to Shandong First Medical University, Jinan, Shandong China

**Keywords:** Blood pressure change, Cerebral small vessel disease, Population-based study, Magnetic resonance imaging

## Abstract

**Objectives:**

This study aimed to investigate the associations between changes in blood pressure (BP) and cerebral small vessel disease (CSVD).

**Methods:**

This study included 401 participants in the magnetic resonance imaging (MRI) sub-study conducted between 2018 and 2020 as a part of the Multidomain Interventions to Delay Dementia and Disability in Rural China project. MRI markers of CSVD were assessed based on international criteria. Individualized linear regression models evaluated changes in BP by estimating the trend of blood pressure changes over time and fitting a straight line from 2014 to 2018. The data were analyzed using logistic and general linear regression models.

**Result:**

The mean age of the participants was 64.48 ± 2.69 years, with 237 (59.1%) being females. Increases in systolic BP in later life were significantly associated with larger volumes of periventricular white matter hyperintensity (WMH), greater perivascular spaces in the basal ganglia (BG-PVS) burden, and the presence of deep lacunes and cerebral microbleeds. Additionally, increases in diastolic BP in later life were significantly associated with the presence of infratentorial and deep lacunes.

**Conclusions:**

CSVDs are associated with increased exposure to elevated BP later in life.

## Introduction

Cerebral small vessel disease (CSVD) poses a significant health concern among the elderly and is often detected through advanced neuroimaging techniques such as magnetic resonance imaging (MRI). The diverse manifestations of CSVD, including white matter hyperintensities (WMH), lacunes, microbleeds, and perivascular spaces (PVS), serve as critical imaging markers for stroke and cognitive impairment in aging individuals [1]. The accessibility of in vivo imaging has experienced rapid advancements, enabling the early detection of cerebrovascular changes. This emphasizes the importance of identifying modifiable risk factors for timely intervention and prevention of stroke and dementia [2].

Among the numerous risk factors associated with cerebrovascular diseases, hypertension (high blood pressure) emerges as a pivotal contributor to the development of stroke, dementia, and CSVD [3]. Recent evidence suggests that changes in blood pressure dynamics over different time frames, ranging from hours to days and years, may play a critical role in influencing the risk of stroke and dementia, irrespective of the absolute blood pressure levels [4–6]. However, the relationship between late-life blood pressure changes and progression of CSVD remains elusive.

Previous research has established a correlation between elevated blood pressure and cerebral small vessel disease (CSVD). Elevated BP variation was associated with a wide range of subclinical brain structural changes, including MRI markers of cerebral small vessel disease, smaller brain tissue volumes, and worse white matter microstructural integrity [7]. Our study aims to delve deeper into this relationship by introducing a novel indicator that not only measures the magnitude, but also the direction of blood pressure fluctuations. In contrast to previous metrics, our methodology offers a comprehensive evaluation of blood pressure variability. We collected blood pressure measurements from 2014 to 2018 for 5 years, aged 60 years and older. An individualized linear model was used to calculate the slope of changes in blood pressure for each older person. Specifically, we fit the blood pressure data of each elderly person to obtain a slope that reflects the trend of blood pressure over time. In the calculation of blood pressure changes, we judge the blood pressure trend of the elderly in recent years by the slope of blood pressure changes. If the slope of the change in blood pressure is positive, it indicates a gradual increase in blood pressure in older people. The greater the absolute value of the slope, the greater the average annual increase in blood pressure. Conversely, if the slope of the blood pressure change is negative, it indicates a downward trend in blood pressure in the elderly. Similarly, the greater the absolute value of the slope, the greater the average annual reduction in blood pressure. In terms of blood pressure measurement, we regularly perform blood pressure measurements on the elderly, ensuring that the measurement is carried out at a fixed time period each year. Before the measurement, the elderly were asked to rest for 5 min, and then the electronic blood pressure monitor (model HEM-7127J) from Omron Corporation in Japan was used to measure the blood pressure of the right upper arm artery in the seated position. Our investigation will specifically focus on examining the correlation between blood pressure change slope and CSVD, a relationship that has received limited attention in prior studies. 

Our study sought to investigate the intricate interplay between changes in blood pressure, as indicated by brain structural imaging biomarkers, and the presence and progression of subclinical brain diseases. We evaluated changes in blood pressure over five years of consecutive visits, considering both their direction and magnitude. By integrating the concept of blood pressure change slope into our analysis, we aspire to deepen our understanding of the influence of blood pressure dynamics on brain health. By fitting the model, we were able to accurately calculate the slope of each participant's blood pressure change based on their specific data. This personalized prediction can better understand the blood pressure trend and change pattern of each person, and is more suitable for monitoring and evaluation of long-term blood pressure trend.

## Method

**Fig. 1:**
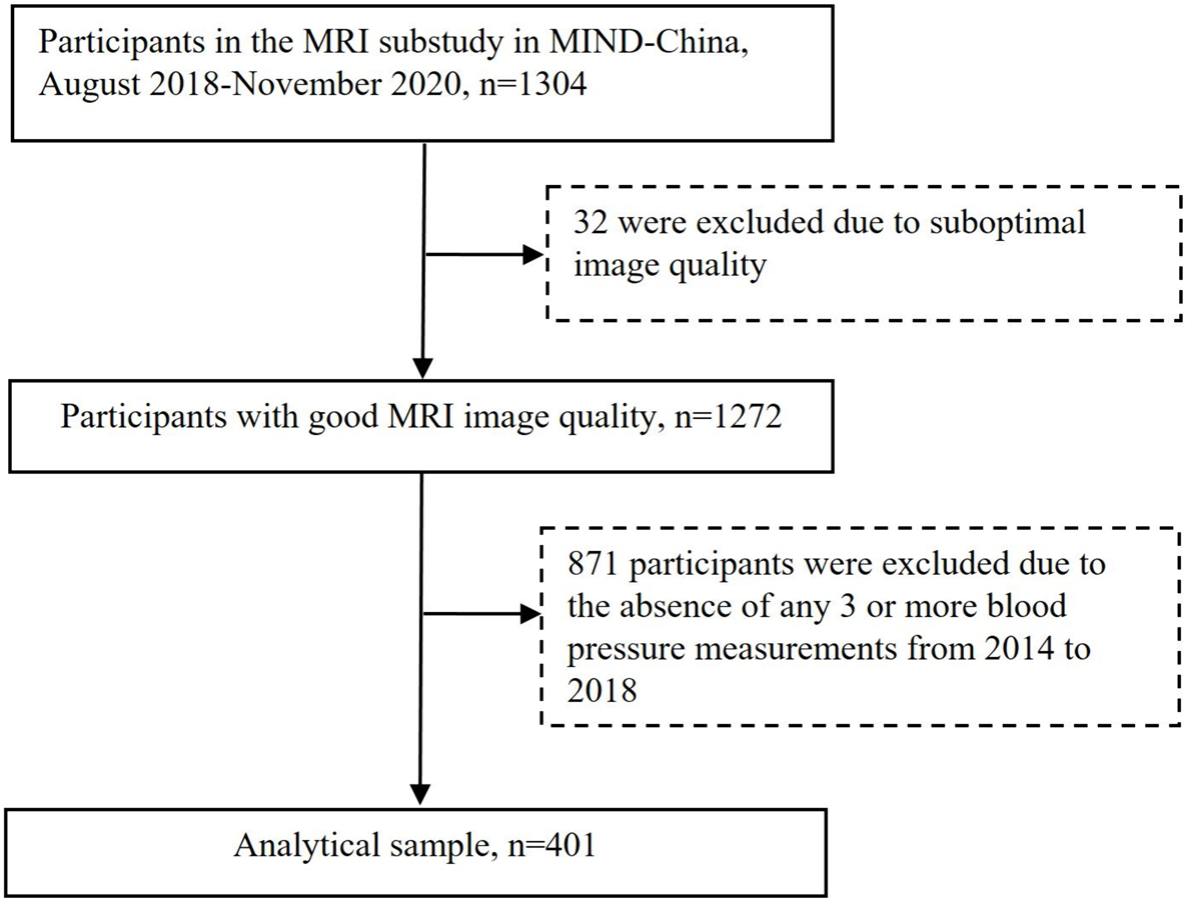
Flowchart of the study participants

This population-based cross-sectional study employed a research design involving participants drawn from the ongoing Multi-Modal Intervention to Delay Dementia and Disability in Rural China (MIND-China) study, a collaborative project of the Global FINGERS network [8]. The MIND-China study’s target population consisted of individuals aged ≥ 60 years residing in 52 villages in Yanlou Township, Yanggu County, western Shandong Province, China. Between March and September 2018, 5765 residents (74.9% of all eligible individuals) underwent baseline assessments, with 1844 participants randomly recruited for a sub-study involving MRI [9]. Among them, 1304 participants completed structural MRI scans between August 2018 and November 2020. Excluding 32 individuals due to suboptimal image quality and missing hypertension assessments on three or more occasions left 401 participants for the current analysis. Figure 1 illustrates the flow of study participants. The MIND-China study received approval from the Shandong Provincial Hospital Ethics Committee in Jinan, Shandong Province, and was conducted in accordance with the ethical principles outlined in the Helsinki Declaration. All participants provided written, informed consent before data collection.

### Data collection and assessments

Trained personnel conducted data collection between March and September 2018 through comprehensive face-to-face interviews, clinical examinations, neuropsychological assessments, and laboratory tests [9, 10]. We employed structured questionnaires to gather extensive data encompassing various domains. These included demographic factors such as age, gender, and education level; lifestyle factors such as alcohol consumption, smoking habits, and physical activity levels; and pertinent health conditions such as hypertension, diabetes, and coronary artery disease. We meticulously documented medication usage, including antihypertensive and antidiabetic drugs, and systematically coded and classified them according to the Anatomical Therapeutic Chemical (ATC) classification system.

Doctors at local town hospitals use commercial electronic blood pressure monitors (Omron HEM-7127J; Omron Corporation, Kyoto, Japan) to measure the arterial blood pressure of all participants [11]. For each blood pressure measurement, the subjects were required to sit still for 5 min so that the cuff was aligned with the heart level. Considering individual differences, physiological factors and measurement errors, so the blood pressure was measured twice, and the average of the two blood pressures was taken as the blood pressure level. Peripheral venous blood was then drawn following an overnight fast. Subsequently, fasting blood glucose, total cholesterol, triglycerides, low-density lipoprotein cholesterol, and high-density lipoprotein cholesterol levels were analyzed using a fully automatic biochemical analyzer (CS-600B, DIRUI Company, Changchun, China) at the laboratory of Yanlou Township Hospital. Participants underwent weight and height measurements while dressed in light attire and without shoes. A 12-lead resting electrocardiogram was recorded with an electrocardiograph machine (CM300, COMEN, Shenzhen, China), and the APOE genotype was determined through a polymerase chain reaction amplification method, classifying individuals as ε4 allele carriers or non-carriers [12]. The education level of the participants was categorized as illiterate (no formal education), primary school, middle school, or higher. Body mass index (BMI) was computed as weight (in kilograms) divided by the square of height (in meters) (kg/m^2^). Smoking and alcohol consumption habits were classified as never smoked and never consumed alcohol.

Hypertension was defined as systolic blood pressure (SBP) ≥ 140 mmHg or diastolic blood pressure (DBP) ≥ 90 mmHg or the current use of antihypertensive medication (ATC codes C02, C03, and C07–C09). Diabetes was defined as fasting blood glucose levels ≥ 7.0 mmol/L, the use of antidiabetic medication (ATC code A10) [13], or a self-reported history of diabetes. Dyslipidemia was characterized by total cholesterol ≥ 6.22 mmol/L, triglycerides ≥ 2.27 mmol/L, low-density lipoprotein cholesterol ≥ 4.14 mmol/L, high-density lipoprotein cholesterol ≥ 1.04 mmol/L, or the use of lipid-lowering agents (ATC code C10). Coronary artery disease, including angina pectoris, myocardial infarction, coronary angioplasty, and coronary artery bypass grafting, was determined based on clinical examinations, self-reported medical history, or electrocardiogram findings. To ensure the late-life blood pressure slope for each participant accurately represented changes in blood pressure in elderly individuals, encompassing both the direction and magnitude of changes in blood pressure, we employed person-specific linear regression models. The blood pressure data of each participant were used as the independent variable, with time as the dependent variable, to estimate the trend of blood pressure changes over time by fitting a straight line, from 2014 to 2018 years. The slope represents the rate of change of blood pressure in late life, i.e., the amount of change per year. Therefore, by calculating the linear regression slope for each participant, we are able to accurately assess their late-life blood pressure changes.

### MRI acquisition and assessment

All eligible participants underwent brain MRI scans utilizing either the Philips Ingenia 3.0T MR system (Philips Healthcare, Best, the Netherlands) at the Southwest Hospital of Shandong University or the Philips Achieva 3.0T MR system (Philips Healthcare, Best, the Netherlands) at the People’s Hospital of Liaocheng. The MRI sequences comprised sagittal 3D T1-weighted, axial T2-weighted, sagittal 3D fluid-attenuated inversion recovery (FLAIR) images, and axial susceptibility-weighted imaging (SWI). Detailed parameters for the core MRI sequences have been previously documented [9]. We assessed MRI biomarkers for four cardiovascular diseases.

Cerebral microbleeds (CMBs) were defined as focal rounded hypointense lesions with a diameter of > 5 mm, as measured on SWI. For quantification, we used AccuBrain^®^ (BrainNow Medical Technology Co., Ltd., Shenzhen, Guangdong, China) [14, 15], employing a fully connected neural network trained in deep learning technology to detect CMBs on SWI images. The network generated a probability map indicating the location of CMBs within the image. The presence of CMBs was defined as a total CMB count of ≥ 1. Visual assessment of lacunes, WMH, and PVS was performed by two trained raters (M.Z. for enlarged PVS [EPVS] and J.W. for lacunes and WMH) following the Standards for Reporting Vascular Changes on Neuroimaging (STRIVE) [16]. Raters, blinded to participants’ clinical data, were supervised by experienced clinical neurologists (L.S.) and neuroradiologists (T.G.). Lacunes were identified as circular or oval lesions with diameters ranging from 3 to 15 mm, showing cerebrospinal fluid signal intensity on T2 and FLAIR sequences. Rater J.W. counted lacunes in different anatomical regions of each hemisphere on FLAIR sequences and then summed the number across both hemispheres. EPVS were identified as small (< 3 mm) punctate (if vertical) or linear (if longitudinal to the scanning plane) high-intensity signals on T2 images, scored using a validated semi-quantitative scale. Rater M.Z. visually counted EPVS in the basal ganglia (BG) and centrum semiovale (CSO) regions on all visible slices and used the number of BG-EPVS on the slice with the most EPVS for analysis.

The total volume of WMH was obtained using the validated AccuBrain^®^ (BrainNow Medical Technology Ltd., Shenzhen, Guangdong, China). T2-FLAIR images were employed to differentiate signal contrast between normal brain tissue and WMH, establishing a signal threshold for WMH recognition. WMH were identified and extracted according to the predetermined threshold using the T2-FLAIR images. The transformed T1-weighted brain structure mask from our study sample was utilized by AccuBrain^®^ to refine and localize the WMH [17].

As previously mentioned, the total CSVD burden was evaluated using an effective CSVD total score. A score of 1 was allocated for the presence of: (a) lacunes; (b) CMBs; and (c) severe EPVS in the BG (> 10). We categorized the total CSVD burden as follows: no CSVD (total CSVD score = 0) and the presence of CSVD (total CSVD score ≥ 1).

### Statistical analysis

Non-parametric Kolmogorov–Smirnov tests were employed to compare age, BMI, PVS count, and volume of WMH. Differences in continuous variables were assessed using the Mann–Whitney U test, while differences in categorical variables were analyzed using the Chi-square test. Binary logistic regression was utilized to examine the relationship between lacunes, CMBs, and the slope of changes in blood pressure. The relationship between PVS and the slope of changes in blood pressure was evaluated using multinomial logistic regression, while ordinary linear regression was used to assess the association between the volume of WMH and the slope of changes in blood pressure. As the following factors were associated with an increased risk of cerebrovascular diseases [18], we adjusted for socio-demographics (age, sex, education), lifestyle behaviors (smoking, alcohol intake), and health conditions (hypertension, diabetes, dyslipidemia, coronary heart disease, stroke and APOE ε4 carrier status). So we present two models: Model 1 adjusted for age, gender, and education level; Model 2 adjusted for BMI, alcohol consumption, smoking, hypertension, diabetes, dyslipidemia, coronary heart disease, and APOE genotype. The statistical analysis was conducted using IBM SPSS Statistics 26.0 for Windows (IBM Corp., Armonk, NY, USA).

## Results

### Characteristics of study participants

Among the 401 participants, the average age was 68.5 years (standard deviation = 2.7), 40.9% were male and 83.8% were presence of cerebral small vessel disease. Compared with non-CSVD, people with CSVD had a higher incidence of disease (hypertension, diabetes, dyslipidemia, coronary heart disease, stroke). Additionally, non-CSVD had lower rates of smoking, alcohol consumption and distribution of the APOE ε4 allele. People in the CSVD group were more likely to have higher BMI than those in the non-CSVD group (*p* < 0.05) (Table 1).

**Table 1:** Characteristics of the study participants by CSVD

Characteristics	Total sample (n = 401)	Non-CSVD (n = 65)	CSVD (n = 336)	*P*-value
Age, years	68.5 (2.7)	67.7 (2.1)	68.6 (2.8)	0.01
Gender, n (%)	164 (40.9) 237(59.1)	25 (38.5) 40 (61.5)	139 (41.4) 197 (58.6)	0.663
Education, n (%)				0.553
Illiterate	120 (29.9.2)	23(35.4)	97 (28.9)	
Primary school	206 (51.4)	30 (46.2)	176 (52.4)	
Middle school or above Hypertension	75 (18.7) 303 (75.6)	12 (18.5) 43 (66.2)	63 (18.8) 260 (77.4)	0.566
Diabetes	62 (15.5)	6 (9.2)	56 (16.7)	0.129
Dyslipidemia	104 (25.9)	30 (46.2)	176 (52.4)	0.566
Coronary heart disease	56 (14.0)	11 (16.9)	45 (13.4)	0.452
Stroke	29 (7.2)	2 (3.1)	27 (8.0)	0.197
Ever smoking	137 (34.2)	21 (32.3)	116 (34.5)	0.452
Ever alcohol intake	150 (37.4)	24 (36.9)	126 (37.5)	0.93
BMI, kg/m^2^	24.65 (3.52)	23.9 (3.5)	24.8 (3.5)	0.048
APOE-4 carrier, n (%)	54 (13.5)	9 (13.8)	45 (13.4)	0.716

*BMI* body mass index, *APOE* apolipoprotein E gene, *WMH* white matter hyperintensity, *PWH* periventricular white matter hyperintensity, *DWH* deep white matter hyperintensity, *BG* basal ganglia, *CSO* centrum semiovale, *EPVS* enlarged perivascular space, *CMBs* cerebral microbleeds

### Associations of slope change of systolic blood pressure in later life with CSVD

**Fig. 2:**
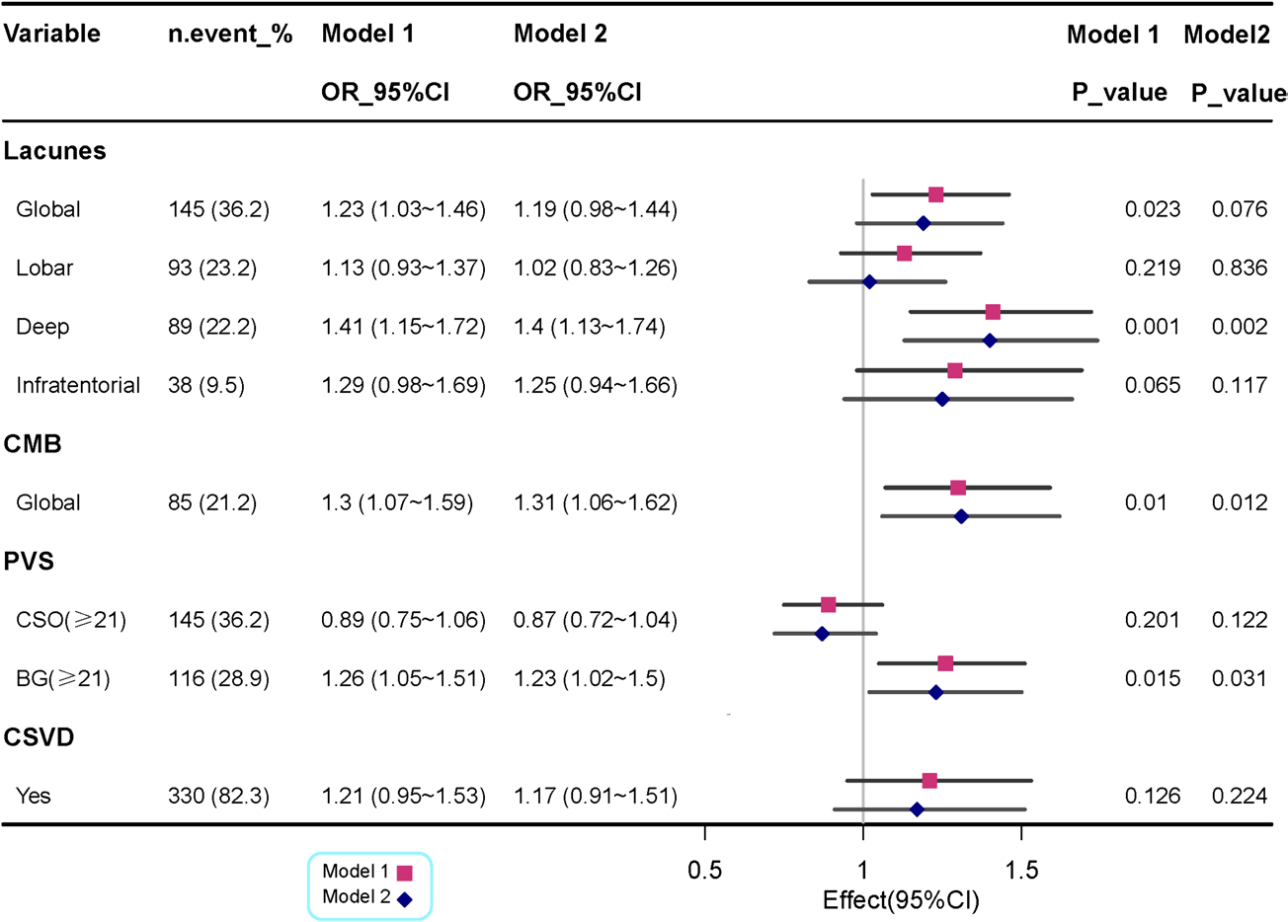
Associations of slope change of systolic blood pressure with cerebral small vessel disease (lacunes, CMB, PVS). Model 1 was adjusted for age, sex, and BMI. Model 2 was further adjusted hypertension, diabetes, dyslipidemia, coronary heart disease, stroke, ever smoking, ever alcohol intake and APOE4 genotype

We used linear and logistic regression models to examine the association of slope change of systolic blood pressure with CSVD MRI markers. After adjusting for demographics, vascular risk factors, and APOE, the slope of late-life SBP change exhibited a significant positive statistical relationship with volumes of both total WMH and PWMHs, but not with DWMH (Table 2). Similarly, in model 1, an increase in the slope of SBP change was significantly associated with both in global lacunes (multi-adjusted odds ratio [OR] = 1.23, 95% confidence interval [CI] 1.03, 1.46; *P* = 0.023) and deep lacunes (multi-adjusted OR = 1.41, 95% CI 1.15, 1.72; *P* = 0.001), however, this association was attenuated and became non-significant in model 2 (Fig. 2, *p* > 0.05). After controlling for multiple potential confounders, the slope of SBP change was positively correlated with global CMBs and a higher BG-PVS burden (Fig. 2).

**Table 2:** Associations of systolic blood pressure change with cerebral small vessel disease (WMH)

WMH	SBP change, β (95%CI)		DBP change, β (95%CI)	
	Model 1	Model 2	Model 1	Model 2
Total WMH volume	0.055 (0.024, 0.087)^*^	0.043 (0.011, 0.074)^*^	0.066 (− 0.019, 0.150)	0.057 (− 0.009, 0.013)
Deep WMH volume	0.012 (− 0.006, 0.030)	0.007 (− 0.012, 0.025)	− 0.005 (− 0.053, 0.043)	− 0.010 (− 0.057, 0.038)
Periventricular WMH volume	0.057 (0.025, 0.090)^*^	0.044 (0.012, 0.077)^*^	0.076 (− 0.009, 0.161)	0.068 (− 0.010, 0.012)

Model 1 was adjusted for age, sex, and BMI. Model 2 was further adjusted hypertension, diabetes, dyslipidemia, coronary heart disease, stroke, ever smoking, ever alcohol intake and APOE4 genotype

*APOE* apolipoprotein E gene

^*^P < 0.05

### Associations of slope change of diastolic blood pressure in later life with CSVD

**Fig. 3:**
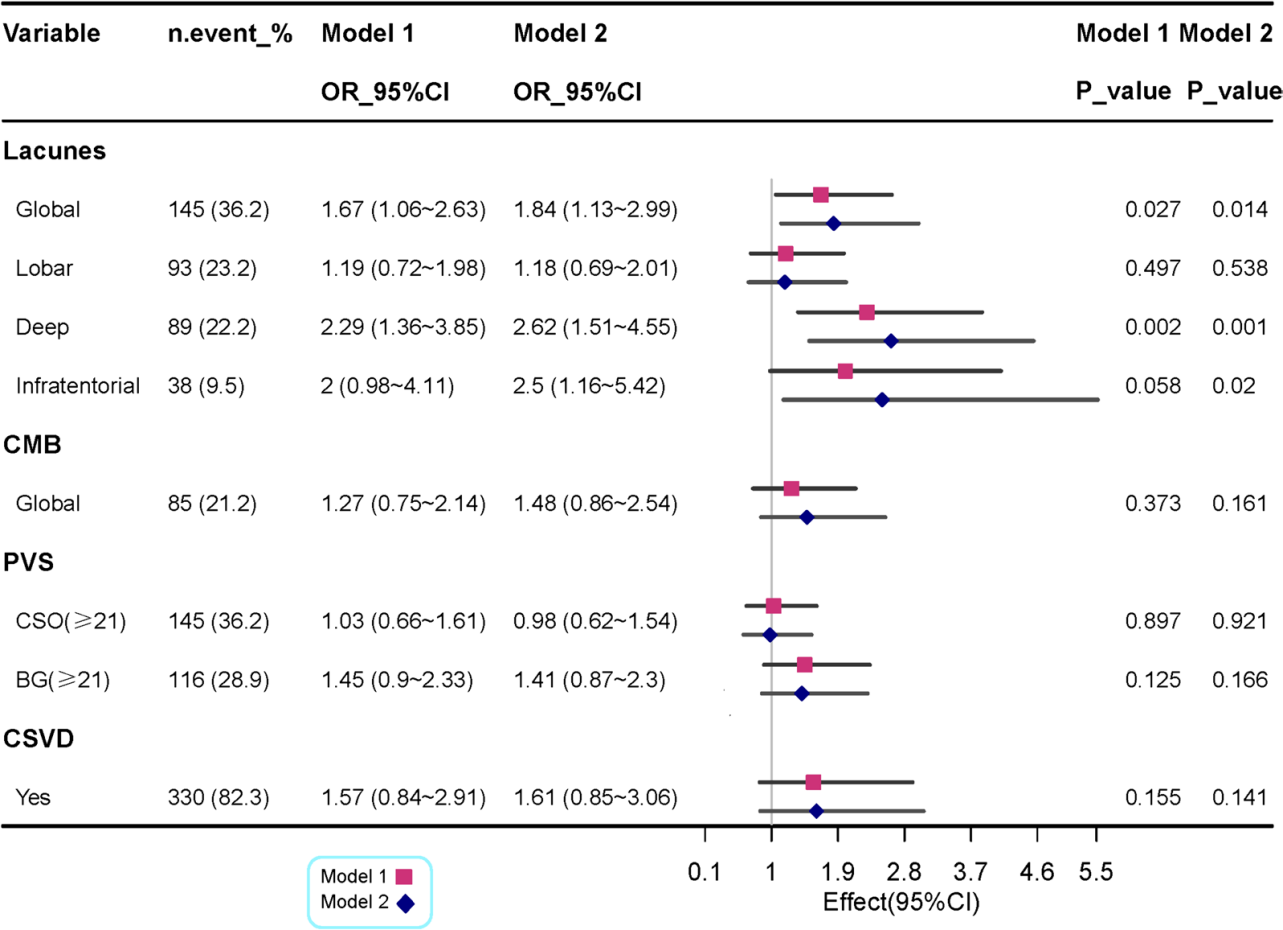
Associations of slope change of diastolic blood pressure with cerebral small vessel disease (lacunes, CMB, PVS). Model 1 was adjusted for age, sex, and BMI. Model 2 was further adjusted hypertension, diabetes, dyslipidemia, coronary heart disease, stroke, ever smoking, ever alcohol intake and APOE4 genotype. Note: APOE, apolipoprotein E gene

Different from SBP change, in the fully adjusted model, no statistically significant relationship was observed between the slope of late-life DBP change and the volume of high signal in the white matter at different sites (Table 2, *p* > 0.05), global CMBs and PVS (Table 2, Fig. 3, *p* > 0.05). In addition, a greater slope of late-life DBP change was significantly associated with a higher global lacunes (multi-adjusted OR = 1.67, 95% CI 1.06, 2.63; *P* = 0.027) and deep lacunes (multi-adjusted OR = 2.29, 95% CI 1.36, 2.63; *P* = 0.002), but not with infratentorial lacunes in model 1 (Fig. 3). While after adjusting for multiple potential confounders in model 2, the slope DBP change was significantly associated with all kinds of lacunes, especially with infratentorial lacunes (multi-adjusted OR = 2.5, 95% CI 1.16, 5.42; *P* = 0.02) (Fig. 3).

## Discussion

Our research findings indicate a significant association between the increased slope of late-life SBP and the total volume of WMHs, PWMHs, deep lacunes, global CMBs, and BG-PVS burden. Additionally, the increased slope of late-life DBP is significantly correlated with whole-brain, deep, and subarachnoid sulcal volumes. These results underscore the importance of considering the direction and magnitude of changes in blood pressure over time, rather than static blood pressure values, in understanding the occurrence and progression of cerebrovascular diseases.

Our study investigated the relationship between the slopes of changes in blood pressure and CSVD, providing better insights into the association between blood pressure variations and brain health [5]. Our findings also contribute new evidence to the relationship between long-term elevated SBP and volume of WMH, consistent with previous reports associating elevated SBP with WMH [6, 7]. The study suggests that the relationship between changes in blood pressure and the presence and progression of WMH appears more pronounced than the relationship with lacunes and CMBs. A logistic regression analysis [19] indicated that both morning SBP and DBP were independent risk factors for total CSVD burden, and the Spearman’s correlation analysis indicated a significant positive correlation between morning SBP and higher deep WMH Fazekas score, EPVS grade in the basal ganglia (*r* = 0.247, *P* = 0.003), and the presence of lacune (*r* = 0.173, *P* = 0.038) and CMB (*r* = 0.326, *P* < 0.001). In line with previous reports [6], the association of BP variation with both the presence and progression of WMH, seemed to be more pronounced compared with lacunes and microbleeds. Increased BP variation was associated with the presence of established markers of cerebral small vessel disease, smaller brain tissue volumes, worse white matter microstructural integrity, and faster progression of WMH [7]. Differences in ethnicity in the population, as well as different cardiovascular and cerebrovascular prevention strategies, may have had different effects on the results of our study.

On one hand, hypertension imposes greater pressure on cerebral vessels, disrupts vascular self-regulation, and elevates the risk of cerebrovascular disease [20, 21]. These mechanisms potentially elucidate the observed association between the steepening trajectory of late-life SBP and the presence of cerebral white matter lesions, CMBs, and an augmented burden of PVS. On the other hand, our findings reveal a significant association between the increased slope of late-life DBP and the total, deep, and subcortical sulcal spaces, indicating the adverse effects of elevated DBP on cerebral vasculature. Elevated DBP may impair vascular dilation function, thereby increasing the susceptibility to various types of cerebrovascular abnormalities. Increased sympathetic activity could lead to increased blood pressure in the early morning, as well as platelet hyperactivation, endothelial cell dysfunction, and increased blood viscosity. Platelet hyperactivation and increased blood viscosity are important factors leading to chronic cerebral ischemia and hypoperfusion. Endothelial cell dysfunction leads to blood–brain barrier disruption and subsequent white matter lesions. Second, morning asthma may promote vascular inflammation, which plays an important role in the pathogenesis and progression of CSVD. These potential mechanisms interact and together contribute to a higher CSVD burden [22].

Elevated SBP results in increased pressure and tension along the arterial walls, fostering the development of atherosclerosis and plaque formation. This process elevates cerebrovascular resistance, diminishes cerebral blood flow perfusion, triggers cerebral tissue hypoxia, and increases the susceptibility to hemorrhagic and ischemic strokes. Concurrently, increased DBP augments arterial resistance, imposes greater cardiac and left ventricular load, and may lead to heart failure [23]. Additionally, elevated DBP increases resistance in small arteries, adversely affecting microcirculation and provoking cerebral tissue ischemia and hypoxia, thereby enhancing the risk of stroke. Fluctuations in blood pressure, whether excessively low or high, can overwhelm the brain’s self-regulatory control mechanisms, potentially leading to vascular damage. Pathophysiologically, chronic hypertension and high blood pressure contribute to increased inflammation and oxidative stress [24, 25], fostering arterial and cerebral atherosclerosis [26], ultimately resulting in lacunar infarcts, white matter lesions, CMBs, and ischemic strokes [27, 28].

Our study has certain limitations that warrant consideration. Firstly, this study relies on self-reported data for health behaviors such as smoking and alcohol consumption, which may introduce recall bias, and the relatively short follow-up period implies that observed associations cannot definitely exclude potential reverse causation. Secondly, the study conclusions may be susceptible to the influence of unaccounted confounding variables, which could affect the interpretation of the findings. Lastly, while the study provides preliminary evidence for an association between the slopes of blood pressure changes and CSVD, it is essential to note that further experimental verification is still needed to establish the causality relationship.

Long-term hypertension change can cause damage to arterioles and venules, resulting in abnormal structure and function of brain micro-vasculature. Hypertension change can lead to lesions in the white matter region of the brain, manifested as white matter hyperintensity. Arteriolar lesions caused by long-term hypertension change may lead to cerebral vascular obstruction or rupture of small vessels, forming lacunar infarction or microhemorrhagic lesions. Effective blood pressure control can reduce the damage of arterioles and microvessels, thus reducing the risk of brain microcirculation disorders and related pathological changes.

Future studies can focus on the impact of different individualized blood pressure control goals (such as age, gender, and basic health status) on the development and progression of CSVD and long-term follow-up studies can be conducted to assess. To explore blood pressure management strategies based on individual characteristics to minimize the risk of CSVD. Through these further studies, we can more comprehensively understand the relationship between blood pressure changes and CSVD and its clinical significance, and provide more effective scientific basis and treatment strategies for improving the prognosis and quality of life of patients with CSVD.

In conclusion, our study offers valuable insights into the association between hypertension and cerebrovascular abnormalities, carrying significant clinical implications for the prevention and treatment of cerebrovascular diseases. Future prospective cohort studies may provide additional insights into the temporal association between late-life blood pressure change and cerebral small vessel disease, which could help in generating hypotheses regarding potential causal mechanisms. Such studies may also provide further evidence for the development of preventive and therapeutic interventions to promote cognitive health in aging and delay the onset of dementia. Future research endeavors should delve deeper into elucidating the mechanisms underlying hypertension's influence on cerebrovascular health and strive to develop tailored treatment approaches for hypertension. Such efforts are crucial for enhancing preventive measures and optimizing the management of cerebrovascular diseases, particularly in elderly patients with hypertension.

## Data Availability

Data will be available on reasonable request.

## Abbreviation

CSVD: Cerebral small vessel disease
BP: Blood pressure
SBP: Systolic blood pressure
DBP: Diastolic blood pressure
MRI: Magnetic resonance imaging
WMH: White matter hyperintensities
PVS: Perivascular spaces
BG: Basal ganglia
CMBs: Cerebral microbleeds
*APOE*: Apolipoprotein E gene
MIND-China: Multidomain Interventions to Delay Dementia and Disability in Rural China
ATC: Anatomical therapeutic chemical
BMI: Body mass index
